# The Cytomegalovirus protein pUL37×1 targets mitochondria to mediate neuroprotection

**DOI:** 10.1038/srep31373

**Published:** 2016-08-26

**Authors:** Chien Tai Hong, Kai-Yin Chau, Anthony H. V. Schapira

**Affiliations:** 1 Department of Clinical Neuroscience, UCL Institute of Neurology, University College London, UK; 2 Department of Neurology, Shuang Ho Hospital, Taipei Medical University, New Taipei City, Taiwan; 3 Department of Neurology, School of Medicine, College of Medicine, Taipei Medical University, Taipei, Taiwan

## Abstract

There is substantial evidence that mitochondrial dysfunction plays a significant role in the pathogenesis of Parkinson disease (PD). This contribution probably encompasses defects of oxidative phosphorylation, mitochondrial turnover (mitophagy), mitochondrial derived oxidative stress, and apoptotic signalling. Human cytomegalovirus immediate-early protein pUL37 × 1 induces Bax mitochondrial translocation and inactivation to prevent apoptosis. Over-expressing pUL37 × 1 in neuronal cells protects against staurosporin and 6-hydroxydopamine induced apoptosis and cell death. Protection is not enhanced by *bax* silencing in pUL37 × 1 over-expressing cells, suggesting a bax-dependent mechanism of action. pUL37 × 1 increases glycolysis and induces mitochondrial hyperpolarization, a bax independent anti-apoptotic action. pUL37 × 1 increases glycolysis through activation of phosphofructokinase by a calcium-dependent pathway. The dual anti-apoptotic mechanism of pUL37 × 1 may be considered a novel neuroprotective strategy in diseases where mitochondrial dysfunction and apoptotic pathways are involved.

Mitochondrial dysfunction is thought to play an important role in the pathogenesis of Parkinson disease (PD) (review by Schapira)^1^. This includes the mitochondrion’s role in oxidative phosphorylation, free radical generation, triggering apoptosis and alteration of mitochondrial turnover (mitophagy). The mitochondrion therefore presents multiple pathways for which to target interventions that could prevent or reverse the deficits and potentially favourably influence the course of PD.

Many viruses, including human cytomegalovirus (CMV), encode proteins that inhibit apoptosis, a powerful innate defence mechanism against viral infection^2^,^3^. UL37 exon 1 protein (pUL37 × 1), which is also known as a viral mitochondria-localized inhibitor of apoptosis, is encoded by the immediate early *ul37* × *1* gene^4^. pUL37 × 1 inactivates Bax and inhibits apoptosis by causing the mitochondrial translocation and conformational change of Bax^5^,^6^,^7^. The Bax-binding function of pUL37 × 1 is essential for the apoptosis prevention, survival of the host cell and replication of the virus. Cells in which the *bax* gene has been silenced are not protected from staurosporin-induced apoptosis.

The anti-apoptotic action of pUL37 × 1 therefore offers a unique and novel mechanism for neuroprotection. We now demonstrate that pUL37 × 1 over-expression protected against toxin-induced cell death and apoptosis in PD cellular models. pUL37 × 1 over-expression protected against cell death and although Bax translocated to mitochondria, apoptosis was prevented. In addition, pUL37 × 1 over-expression also increased cellular glycolysis and hyperpolarized mitochondria, actions which contributed to the neuroprotective mechanism of pUL37 × 1 in these models.

## Results

### Generation and Characterization of Three pUL37 × 1 Over-expressing SH-SY5Y Cell Lines

In order to evaluate the neuroprotective potential of pUL37 × 1 over-expression, p*ul37* × *1* cDNA fused with a 3′ haemagluttinin (HA) epitope was cloned into pcDNA3.1 plasmid and transfected into SH-SY5Y cells. Three independent stable over-expressing lines, named as pUL37 × 1-1 to 3 in the following paragraphs were obtained upon geneticin selection. Control lines used in this study included SH-SY5Y cells (labelled as control-1), SH-SY5Y cells over-expressing dsRed in the mitochondria (control-2) and SH-SY5Y cells with pcDNA3.1(+) plasmid (control-3). Data which were labelled as control and pUL37 × 1 were the combination of each cell line except where specified.

**Figure 1:**
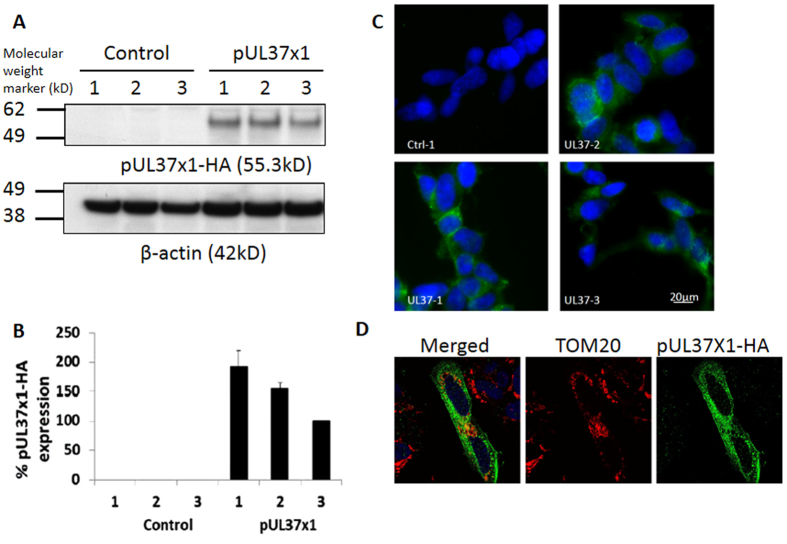
Generation and characterization of stable pUL37 × 1 over-expressing SH-SY5Y cell lines. (**A**) Three independent pUL37 × 1 over-expressing SH-SY5Y cell lines were generated and analyzed for this study. Representative Western blot image demonstrates the expression of pUL37 × 1-HA. pUL37 × 1-HA was detected by anti-HA antibody. Control- line 1 was normal SH-SY5Y cells; control-line 2 was SH-SY5Y cell over-expressing dsRed-Mito and control-line 3 was SH-SY5Y cells with the empty vector, pcDNA3.1(+). β-actin was loading control. (**B**) Densitometry analysis of pUL37 × 1 expression level: pUL37 × 1 line-1 expressed the most (192.2 ± 27.7%), followed by line-2(155.4 ± 9.2%) and line-3 (100 ± 0%)(n = 6). The expression level was normalized by line-3 and corrected by β-actin. Data were presented as mean ± S.E.M. The experiments had been repeated for three times. (**C**) All three over-expressing lines homogenously expressed pUL37 × 1-HA which was detected by immunocytochemistry whereas the control SH-SY5Y cells did not express detectable pUL37 × 1-HA (green: pUL37 × 1-HA, blue: DAPI). pUL37 × 1-HA was detected by anti-HA antibody. (**D**) The mitochondrial localization of pUL37 × 1. In the representative image, mitochondria were labelled by TOM20 (red) and pUL37 × 1-HA was detected by anti-HA antibody (green). In the merged image, a substantial co-localization is demonstrated (yellow).

The representative Western blot image confirmed that the selected over-expressing cell lines expressed pUL37 × 1-HA by producing a distinctive band of size equivalent to the calculated molecular weight of 55.3 kDa detected by anti-HA antibody (Fig. 1A), which was absent from the three different control lines. pUL37 × 1-1 expressed the most pUL37 × 1 followed by pUL37 × 1–2 and pUL37 × 1-3 (Fig. 1B). The purity of ectopic expression in each line was confirmed by immunocytochemistry, which showed that most of the cells in pUL37 × 1 over-expressing lines exhibited positive pUL37 × 1-HA staining (Fig. 1C). Immunocytochemistry and confocal microscopy confirmed a substantial mitochondrial localization of pUL37 × 1 (Fig. 1D). pUL37 × 1-HA was detected by anti-HA.

### pUL37 × 1 Over-expression Protected Against Toxin-Induced Apoptosis and Cell Death

**Figure 2:**
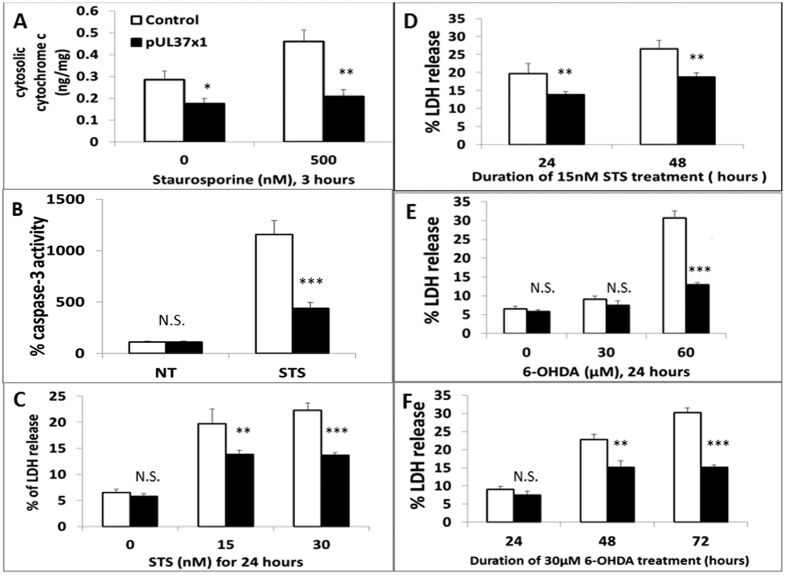
pUL37 × 1 over-expression protected against toxin-Induced apoptosis and cell death in SH-SY5Y cells. (**A**) pUL37 × 1 over-expression significantly reduced cytosolic cytochrome c upon 500 nM staurosporine treatment for 3 hours (0.46 ± 0.05 ng/mg cell lysate in control versus 0.21 ± 0.03 ng/mg cell lysate in pUL37 × 1 over-expression lines, *p* < 0.01, n = 3). (**B**) pUL37 × 1 over-expression did not alter the baseline caspase-3 activity (set as 100%) but significantly lowered the increase of caspase-3 activity upon 500 nM staurosporine treated for 4 hours (1156.5 ± 137.2% in control versus 440.5 ± 56.3% in pUL37 × 1 over-expression, *p* < 0.001, n = 5). (**C**) pUL37 × 1 over-expression did not increase spontaneous cell death compared with control. pUL37 × 1 over-expression significantly protected cell death upon either 15 or 30 nM staurosporine (STS) treatment for 24 hours (15 nM: 25.4 ± 2.3% in control, 13.9 ± 0.8% in pUL37 × 1 over-expression, *p* < 0.01, n = 4; 30 nM: 22.3 ± 1.3% in control, 13.7 ± 0.5% in pUL37 × 1 over-expression, *p* < 0.001, n = 8). (**D**) pUL37 × 1 over-expression significantly reduced the percentage of LDH release induced by 15 nM staurosporine treatment for 24 and 48 hours (24 hours: 25.4 ± 2.3% in control, 13.9 ± 0.8% in pUL37 × 1 over-expression, *p* < 0.01, n = 4; 48 hours: 22.8 ± 1.5% in control, 15.1 ± 1.9% in pUL37 × 1 over-expression, *p* < 0.01, n = 6). (**E**) pUL37 × 1 over-expression significantly decreased the percentage of LDH release induced by 60 μM 6-OHDA treatment for 24 hours (30.6 ± 1.9% in control, 12.9 ± 0.6% in pUL37 × 1 over-expression, *p* < 0.001, n = 6). (**F**) pUL37 × 1 over-expression significantly decreased the percentage of LDH release induced by 30 μM 6-OHDA treatment for 48 and 72 hours (48 hours: 22.8 ± 1.5% in control, 15.1 ± 1.9% in pUL37 × 1 over-expression, *p* < 0.01, n = 8; 72 hours: 30.2 ± 1.3% in control, 15.1 ± 0.6% in pUL37 × 1 over-expression, *p* < 0.001, n = 6). Data were presented as mean ± S.E.M. Statistics was performed by two-tailed Student’s t- test. The n numbers represented the experimental repeat of each pUL37 × 1 over-expressing and control lines (NS, non-significant, *p < 0.05, **p < 0.01; ***p < 0.001).

Cytochrome c, an electron carrier between complex III and IV, is also known to induce apoptosis when it is released into the cytoplasm. pUL37 × 1 over-expression significantly prevented the release of cytochrome c from mitochondria to cytoplasm. Staurosporine is a protein kinase C inhibitor and apoptosis inducer; 500 nM treatment for 3 hours caused increased cytochrome c release in the control which was blocked in pUL37 × 1 over-expressing cells (Fig. 2A). In addition, pUL37 × 1 over-expression significantly reduced the cytosolic cytochrome c in the absence of toxin.

The activation of caspase-3 is an important component of the apoptosis pathway. pUL37 × 1 over-expression was expected to prevent the activation of caspase-3 due to blocking of the upstream apoptosis process. In the absence of toxin, pUL37 × 1 over-expression did not affect the activity of caspase-3 compared to control. However, following 500 nM staurosporine treatment for 4 hours, pUL37 × 1 over-expressing SH-SY5Y cells exhibited significantly lower caspase-3 activity compared to control (Fig. 2B). Based on these findings, pUL37 × 1 over-expression is able to block the apoptosis cascade induced by staurosporine.

pUL37 × 1 over-expression also protected against cell death induced by staurosporin and 6-hydroxydopamine (6-OHDA). 6-OHDA is an oxidative stress inducer and widely-used in PD *in vivo* and *in vitro* models^8^. Stable pUL37 × 1 over-expressing cell lines did not show increased spontaneous cell death compared to control, as determined by LDH release (Fig. 2C). pUL37 × 1 over-expression significantly reduced the cell death induced by 30 nM staurosporin treatment for 24 hours and 15 nM staurosporin treatment for 24 or 48 hours (Fig. 2C,D). Moreover, pUL37 × 1 over-expression also protected against 60 μM 6-OHDA treatment for 24 hours and 30 μM 6-OHDA for 48 and 72 hours treatment (Fig. 2E,F). Similar effects were observed using propidium iodide (PI) staining assay (Supplement, S1). The levels of protection were similar in all three pUL37 over-expressing lines (Supplement, S2).

### pUL37 × 1 Over-expression Protected against Apoptosis and Neuronal Death in Rat Primary Cortical Cultures

Primary rat cortical cultures were used to investigate further the protection conferred by pUL37 × 1 over-expression. Liposomal transfecting reagent mixed with red fluorescence protein (RFP) plasmid (control) or a mixture of pUL37 × 1 and RFP plasmids at 10 to 1 ratio (experiment) were co-incubated with the cultures for 48 hours. pUL37 × 1 over-expression was identified by the expression of RFP.

**Figure 3:**
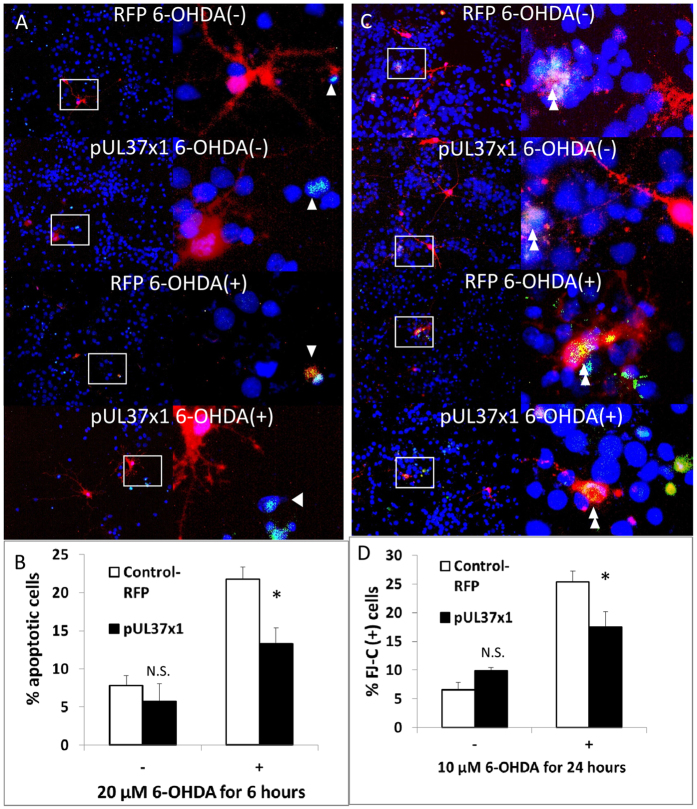
pUL37 × 1 over-expression protected against apoptosis and neuronal death in rat primary cortical culture. (**A**) The representative images of apoptotic cell counting by positive cleaved caspase-3 stain (green) on RFP and pUL37 × 1 transfecting rat primary cortical culture. Because the ratio of concentration between pUL37 × 1 and RFP is 10:1, expression of RFP (red) is assumed as pUL37 × 1 expressing neurons. The apoptotic neurons were identified based on the RFP-expressed cells with positive cleaved caspase-3 stain (arrowhead) whereas RFP-expressed cells without cleaved caspase-3 stain were recognized as survival neurons. Low magnification images (left) were used to evaluate incidents of apoptosis whereas high magnification images (right, corresponding to the boxed area in the low magnification images) show the representative immunoreactivity of cleaved caspase-3 on transfected neurons. For each rat cortical culture preparation, at least ten low magnification images were taken to quantitative analysis. (**B**) pUL37 × 1 expression significantly reduced the percentage of apoptotic cells induced by 20 μM 6-OHDA treatment for 6 hours compared with RFP expressing ones (21.8 ± 1.6% in RFP-control versus 13.3 ± 2.1% in pUL37 × 1 over-expression, *p* < 0.05, n = 6). (**C**) The representative images of fluoro-jade C (FJ-C) stain. Similar to the detection of apoptotic neurons, RFP-expressed neurons (red) with positive FJ-C stain (green, double arrowhead) were counted as death neurons whereas RFP-expression only neurons were survival neurons. Low magnification images (left) were used for counting dead neurons whereas high magnification images (right, corresponding to the boxed area in the low magnification images) show the representative fluorescent FJ-C staining. (**D**) pUL37 × 1 expression significantly reduced the percentage of FJ-C positive neurons induced by 10 μM 6-OHDA treatment for 24 hours compared with RFP expressing ones (25.3 ± 1.9% in RFP-control versus 17.4 ± 2.7% in pUL37 × 1 over-expression, *p* < 0.05, n = 6). Data were presented as mean ± S.E.M. Statistics was performed by two-tailed Student’s t- test. (NS, non-significant, *p < 0.05 ).

The anti-apoptotic effect of pUL37 × 1 over-expression on primary rat cortical culture was detected by the antibody against cleaved caspase-3, which is the activated form of caspase-3. As expected, 6-OHDA treatment resulted in a significant increase in apoptosis in cortical neurons. pUL37 × 1 over-expression alone did not increase apoptosis compared to control. However, following 20 μM 6-OHDA treatment for 6 hours, pUL37 × 1 over-expression significantly reduced the percentage of apoptotic cells(Fig. 3A,B). Similar results were obtained using Fluoro-Jade C (FJ-C), as a specific marker for degenerative neurons. pUL37 × 1 over-expression significantly reduced the neuronal death labelled by FJ-C compared with control following 10 μM 6-OHDA treatment for 24 hours (Fig. 3C,D). The transfection efficiency was approximately 5%.

### The protection of pUL37 × 1 is Bax-dependent

The mitochondrial sequestration and inactivation of Bax has been postulated as an anti-apoptotic mechanism of pUL37 × 1^5^,^6^,^7^. In the present study, pUL37 × 1 over-expressing cells exhibited some co-localization of Bax with mitochondria, supporting a mitochondrial translocation of Bax (Supplement S3A).

**Figure 4:**
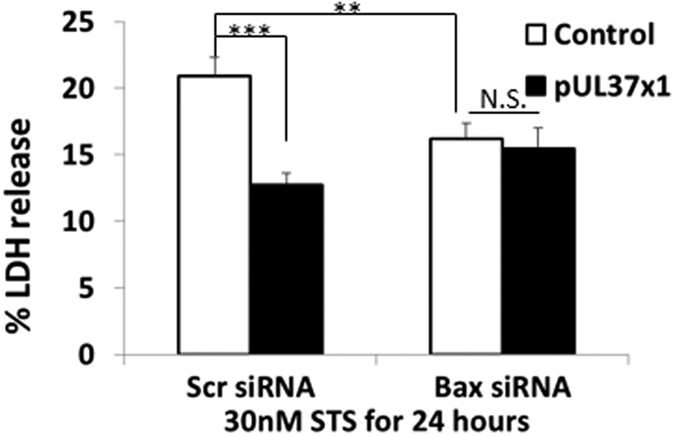
The protection of pUL37 × 1 over-expression resulted from the Bax inactivation. 5 nM *bax* siRNA silencing for 3 days significantly reduced LDH release upon 30 nM staurosporine treated for 24 hours (20.94 ± 1.35% in scramble siRNA versus 16.19 ± 1.09% in *bax* siNRA, *p* < 0.01, n = 8). However, this reduction did not present in pUL37 × 1 over-expressing SH-SY5Y cell. In addition, there was no significant difference of LDH release upon 30 nM staurosporine treated for 24 hours between controls with pUL37 × 1 over-expressing SH-SY5Y cells when both of them underwent 5 nM *bax* siRNA silencing. Data were presented as mean ± S.E.M. Statistics was performed by two-tailed Student’s t- test. The n number represented the experimental repeat from each pUL37 × 1 over-expression lines and control (include normal SH-SY5Y cells, over-expressing dsRed-mito SH-SY5Y cells and over-expressing pcDNA Zero SH-SY5Y cells) (NS, non-significant, **p < 0.01; ***p < 0.001).

To investigate this protective mechanism further, both control and pUL37 × 1 over-expressing cells were treated with either scrambled siRNA or *bax* siRNA for 3 days. *bax* siRNA silencing substantially reduced the expression of Bax compared with scrambled siRNA (Supplement S3B). Following challenge with 30 nM staurosporin for 24 hours, *bax* siRNA silencing reduced the percentage of LDH release compared with scrambled siRNA silencing in control cells. In the presence of scrambled siRNA silencing, pUL37 × 1 over-expression remained protective against staurosporin. However, the combination of *bax* siRNA silencing with pUL37 × 1 did not modify LDH release (Fig. 4). These data indicate that the anti-apoptotic effect of pUL37 × 1 over-expression was dependent on Bax activity.

### Glycolysis-induced Mitochondrial Hyperpolarization as a Protective Mechanism of pUL37 × 1

**Figure 5:**
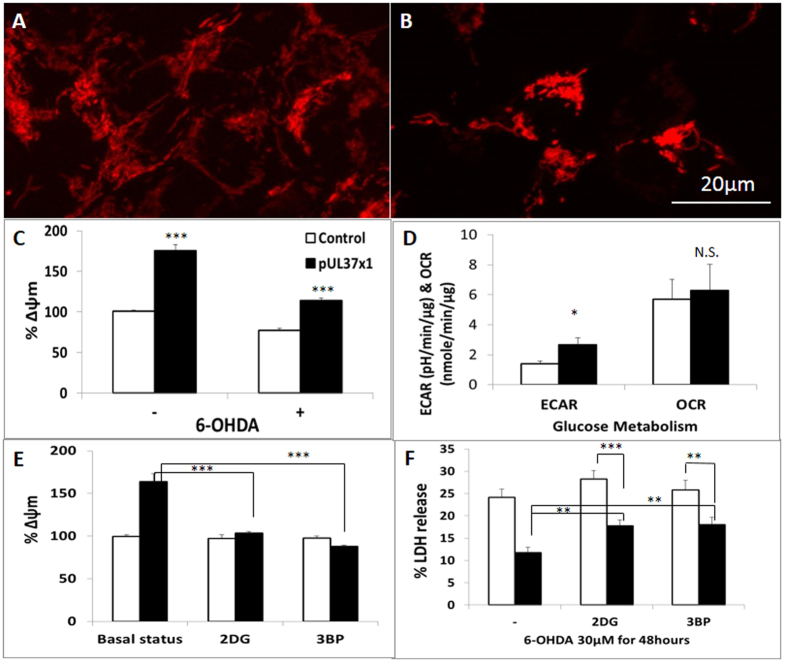
The protection of pUL37 × 1 over-expression is mediated by mitochondrial hyperpolarization and glycolysis. (**A**,**B**) The representative images of TMRM staining from control (**A**) and pUL37 × 1 over-expressing cell (**B**). pUL37 × 1 over-expressing cell exhibits higher TMRM staining comparing with control. Both control and pUL37 × 1 over-expressing cells were assessed under identical condition, exposure time and intensity gain (**C**) The fluorescence intensity of TMRM was calculated by ImageJ with adjustment of mitochondrial content. pUL37 × 1 over-expression significantly hyperpolarized baseline mitochondrial membrane potential (175.3 ± 7.4% compared with 100.0 ± 1.4% from control, *p* < 0.001, n = 24 from control SH-SY5Y cells and n = 8 from each pUL37 × 1 over-expressing line) and rescued the mitochondrial depolarization induced by 100 μM 6-OHDA treatment for 1 hour (114.1 ± 3.2% compared with 77.0 ± 2.4% from control, *p* < 0.001, n = 10 from control SH-SY5Y cells and n = 5 from each pUL37 × 1 over-expressing line). (**D**) pUL37 × 1 over-expression induced a significant increase of extracellular acidification rate (ECAR) compared with control (pUL37 × 1: 2.68 ± 0.46, control: 1.39 ± 0.20 pH/min/μg, *p* < 0.05, n = 10) without impaired oxidative phosphorylation as measure by oxygen consumption rate (OCR). (**E**) Glycolytic inhibitors, either 10 μM 2-deoxy-glucose (2DG) or 5 μM 3-Bromopyruvate (3BP), significantly reversed the phenomenon of mitochondrial hyperpolarization resulted from pUL37 × 1 over-expression (pUL37 × 1: 163.6 ± 9.3%, pUL37 × 1 + 10 μM 2DG: 103.7 ± 1.9%, pUL37 × 1 + 5 μM 3BP: 87.8 ± 2.8%, *p* < 0.001 respectively, n = 10). (**F**) In the presence of glycolytic inhibitors, the protection of pUL37 × 1 over-expression against 30 μM 6-OHDA, 48 hours treatment was still significant but attenuated (pUL37 × 1: 11.7 ± 1.2%, pUL37 × 1 + 10 μM 2DG: 17.8 ± 1.3%, pUL37 × 1 + 5 μM 3BP: 18.0 ± 1.6%, *p* < 0.01 respectively, n = 6). Data were presented as mean ± S.E.M. Statistics was performed by either one-ANOVA with Dunnett’s post-hoc analysis or Student’s t-test. (N.S., non-significant, *p < 0.05, **p < 0.01, ***p < 0.001).

Mitochondrial membrane depolarization is a common phenomenon during apoptosis^9^, and has been reported to take place before Bax translocation^10^. Hyperpolarization of mitochondria has been shown to be a protective strategy^11^. pUL37 × 1 over-expression induced hyperpolarization of mitochondria in SH-SY5Y cells (Fig. 5A,B). This hyperpolarization was reduced but was still significantly above control following exposure to 6-OHDA (Fig. 5C).

Mitochondrial hyperpolarization can result from complex V inhibition, insufficient ADP supply or increased glycolysis^12^. However, the two former result in energy deficit and lead to cell death, which did not occur at baseline in pUL37 × 1 over-expression (Fig. 5C). CMV infection has been found to increase glycolysis in host cells. pUL37 × 1 over-expression significantly increased glycolysis measured by the XF analyser compared with control in SH-SY5Y cells (Fig. 5D). This glycolysis-enhancing effect did not result from oxidative phosphorylation (OXPHOS) inhibition as there was neither significant difference in oxygen consumption rate (OCR) between control and pUL37 × 1 over-expressing SH-SY5Y cells (Fig. 5D), nor reduced ADP phosphorylation and enzymatic activity from each mitochondrial respiratory chain complex (Supplement S4).

Glycolytic inhibitors, either 2-deoxyglucose (2DG) and 3-bromopyruvate (3BP) were applied to pUL37 × 1 over-expressing SH-SY5Y cells to verify glycolysis-dependent mitochondrial hyperpolarization. In control SH-SY5Y cells, neither 10 μM 2DG nor 5 μM 3BP altered the mitochondrial membrane potential. However, the hyperpolarization induced by pUL37 × 1 over-expression dissipated following treatment with either 2DG or 3BP (Fig. 5E). This result confirmed that the mitochondrial hyperpolarization associated with pUL37 × 1 over-expression was dependent on its glycolysis-enhancing effect.

To determine whether the glycolytic-dependent mitochondrial hyperpolarization contributes to the protective effect of pUL37 × 1 we assessed the effect of glycolysis inhibition with toxin exposure. Both 10 μM 2DG and 5 μM 3BP attenuated but did not prevent the protective effect of pUL37 × 1 over-expression against 6-OHDA (Fig. 5F).

### pUL37 × 1 over-expression Increased Glycolysis is Calcium Dependent

**Figure 6:**
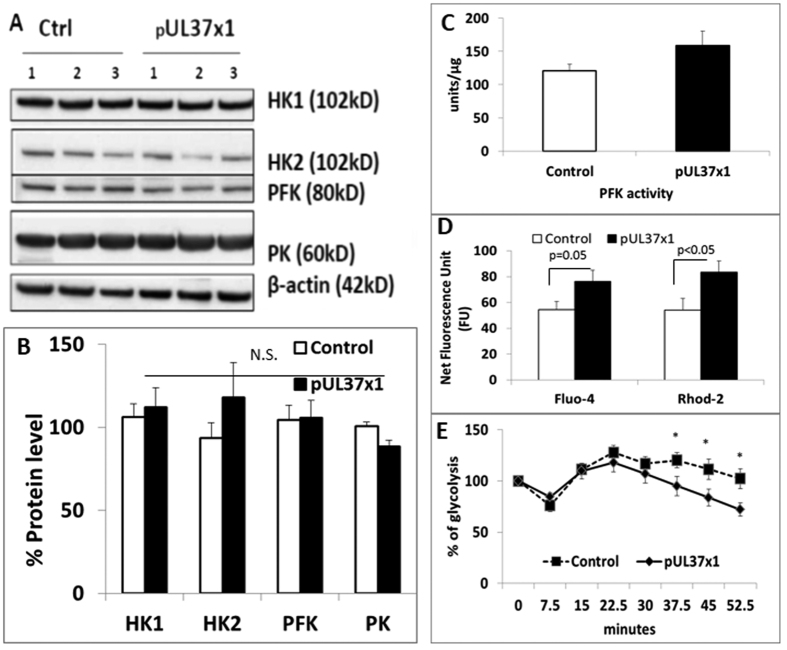
That pUL37 × 1 increased glycolysis was dependent to intracellular calcium. (**A,B**) Representative western blot and densitometry analysis revealed that pUL37 × 1 over-expression did not alter the protein level of certain key glycolytic enzymes, including hexokinase 1 (HK1), hexokinase 2 (HK2), phosphofructokinase (PFK) and pyruvate kinase (PK) on SH-SY5Y cells. The expression level was corrected by the level of β-actin, which served as a loading control (n = 3). (**C**) pUL37 × 1 over-expression increased phosphofructokinase (PFK) activity. (pUL37 × 1: 158.4 ± 22.4, control: 121.0 ± 9.9 units/μg, n = 6) (**D**) pUL37 × 1 over-expression increased intracellular and mitochondrial calcium. The net fluorescence unit (FU) of Fluo-4 and Rhod-2 were higher in pUL37 × 1 over-expressing cells compared with control (FU of Fluo-4: control :54.5 ± 6.2, pUL37 × 1: 76.4 ± 8.5; FU of Rhod-2: control: 54.1 ± 9.0, pUL37 × 1: 83.4 ± 8.6). (**E**) Upon 30 nM BAPTA-AM treatment, the glycolytic activity of pUL37 × 1 over-expressing cells was significantly lower than control at 37.5 (control: 120.2 ± 7.5%, pUL37 × 1:95.1 ± 9.3%), 45 (control: 111.5 ± 9.9%, pUL37 × 1:83.8 ± 8.0%), and 52.5 (control: 102.3 ± 9.7%, pUL37 × 1:72.1 ± 6.6%) minutes time point. n = 4. The glycolytic activity was normalized by basal glycolytic activity from each group (pUL37 × 1 and control). Data were presented as mean ± S.E.M. Statistics was performed by two-tailed Student’s t- test. (N.S., non-significant, *p < 0.05).

The rate of glycolysis is normally tightly controlled by three enzymes: hexokinase (HK), phosphofructokinase (PFK) and pyruvate kinase (PK). Increased glycolytic metabolites and flux in host cells have been noted during CMV infection^13^,^14^. In the present study, there was no significant difference in the expression levels of these proteins between control and pUL37 × 1 over-expressing cells (Fig. 6A,B). However, pUL37 × 1 over-expression up-regulated PFK enzymatic activity (Fig. 6C). This suggests that pUL37 × 1 modulates glycolysis through a post-translational mechanism.

The activity of PFK is tightly regulated by intracellular calcium levels: high intracellular calcium triggers the binding of calmodulin with PFK with formation of the activated dimer of the enzyme^15^. pUL37 × 1 is known to cause the release of calcium from endoplasmic reticulum (ER)^16^, which may account for the enhanced PFK activity and increased glycolysis. The present study demonstrated that pUL37 × 1 over-expression increased both intracellular calcium (measure by Fluo-4) and mitochondrial calcium (measure by Rhod-2) (Fig. 6D). The increase of both intracellular and mitochondrial calcium indicated the release of calcium from ER. In order to establish the relationship between glycolysis and intracellular calcium level, BAPTA-AM, a cell permeable calcium caging agent was used to investigate the causal effect. After 30 nM BAPTA-AM treatment for 50 minutes, glycolytic activity was significantly reduced in pUL37 × 1 over-expression cells compared with baseline, with no change in control cells (Fig. 6E). These results indicate that pUL37 × 1 up-regulates glycolysis through a calcium-dependent pathway to activate PFK.

## Discussion

**Figure 7:**
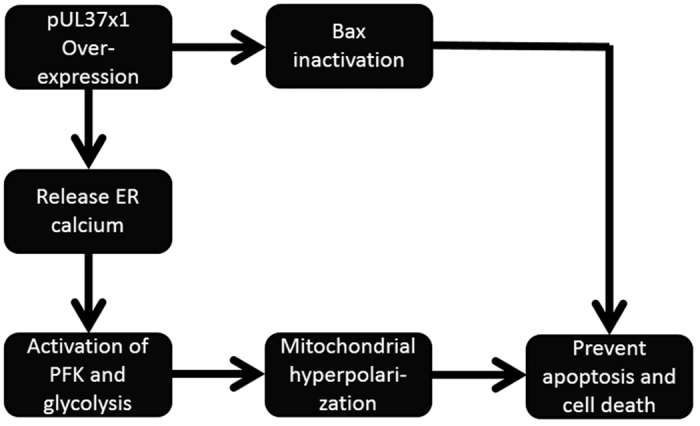
Summary of dual anti-apoptotic effects of pUL37 × 1. In the present study, we demonstrated that pUL37 × 1 over-expression protected apoptotic death through two pathways: one protective mechanism of pUL37 × 1 depended on Bax inactivation and the other was due to mitochondrial hyperpolarization. pUL37 × 1 over-expression induced the increased intracellular calcium, which activated phosphofructokinase (PFK) and up-regulated glycolysis, followed by mitochondrial hyperpolarization.

The present study demonstrated the neuroprotective effect of pUL37 × 1 over-expression against staurosporin and 6-OHDA-induced apoptosis and cell death in both SH-SY5Y and primary rat cortical cultured cells. This protective effect of pUL37 × 1 appears to be mediated through two separate mechanisms, both of which modulate mitochondria-dependent apoptosis. pUL37 × 1, located on the mitochondrial outer membrane, led to the mitochondrial translocation and inactivation of Bax. The second mechanism involved mitochondrial hyperpolarization with increased calcium release and enhanced PFK-mediated glycolysis and hyperpolarization. Cell death protection was attenuated with inhibitors of glycolysis and of calcium release (summary in Fig. 7). The strategy of using viral derived proteins as a means to target mitochondria for neuroprotection has recently been supported by using a protein derived from Borna disease virus^17^and the β-2.7 RNA from CMV^18^.

The advantages of using stable pUL37 × 1 over-expressing lines instead of transient transfection include long-term gene expression, consistency and the option of further genetic manipulation^19^. After antibiotic selection and pUL37 × 1-HA expression confirmation by western blot analysis, we selected three lines in order to eliminate the impact of possible cloning artefacts. All three lines presented a homogenous pUL37 × 1-HA expression by immunocytochemistry, which also confirmed the mitochondrial localization of pUL37 × 1 in SH-SY5Y cells. This has been reported previously in HeLa cells^20^ and the first domain of pUL37 × 1 has been identified as responsible for mitochondria targeting and localization^21^.

The release of cytochrome c from mitochondria to cytoplasm results from mitochondrial membrane permeabilization (MMP), is a point-of-no-return in apoptosis. Cytoplasmic cytochrome c interacts with Apaf-1 protein to form an apoptosome and activates caspase. Hence, both the release of cytochrome c and the activation of caspase, especially caspase-3, are the hallmarks of apoptosis processing^22^. pUL37 × 1 over-expression in SH-SY5Y and primary rat cortical cells reduced the release of cytochrome c and activation of caspase-3 and decreased cell death upon staurosporin and 6-OHDA treatment. It is still important to measure cell death protection after confirmation of anti-apoptotic effect because in some occasions, down-regulation of apoptosis can promote necrosis^23^,^24^. In SH-SY5Y cells, the protection was present under several different conditions, including high dose, short-term toxin treatment and moderate dose, longer-term toxin treatment. These results indicate that pUL37 × 1 is anti-apoptotic in neuronal cells and neuroprotective in cellular models.

Bax, a pro-apoptotic bcl-2 family protein, is thought to play an important role in apoptotic neuronal death in PD^25^,^26^. Bax ablation has been demonstrated to have an anti-apoptotic and neuroprotective effect. Vila *et al*. show that Bax deficient mutant mice were resistant to MPTP neurotoxicity^27^. In the present study, *bax* siRNA silencing reduced Bax protein expression levels and resulted in cell death protection against staurosporin in control cells. However, silencing of *bax* in pUL37 × 1 over-expressing cells did not provide further cell death protection against staurosporin compared with pUL37 × 1 expressing cells. Failure of cell death protection by *bax* siRNA silencing in pUL37 × 1 over-expressing cells indicated a Bax-dependent neuroprotective mechanism of pUL37 × 1, which support the Bax inactivation effect of pUL37 × 1 from previous reports^5^,^6^,^7^.

Mitochondria can become hyperpolarized due to the reverse of proton trafficking through complex V. When the intracellular ATP is sufficient, complex V consumes ATP to pump protons out rather than in. The main source of extra-mitochondrial ATP generation is glycolysis. Glycolysis is an alternative energy resource to oxidative phosphorylation, and though it might be seen as bioenergetically inefficient, due to low ATP and NADP yield, because of its fast reaction rate, glycolysis is able to provide sufficient energy to meet cellular demand^28^. Cancer cells are thought to generate ATP predominantly from glycolysis even in oxygen-rich conditions, the Warburg effect^29^. Owing to this alteration of glucose metabolism, cancer cells usually contain hyperpolarized mitochondria and are less sensitive to apoptosis^30^,^31^,^32^. Cells with higher glycolytic capacity, such as astrocytes and myocytes, also utilize glycolysis to protect cell death by enhancing mitochondrial membrane potential^33^,^34^. In *in vitro* PD models, higher glycolysis activity contributed to neuroprotection against MPTP^35^,^36^,^37^. We have demonstrated a novel protective effect of pUL37 × 1 that is mediated via the enhancement of glycolysis and leads to the hyperpolarization of the mitochondrial membrane potential. Glycolytic inhibitors attenuated the hyperpolarization and protective effects of pUL37 × 1 over-expression. Mitochondrial hyperpolarization also resulted in reduced cytosolic cytochrome c level in the absence of toxin. In physiological status, cytochrome c is loosely attached to the mitochondrial inner membrane due to the presence of mitochondrial membrane potential. Hyperpolarization of mitochondrial membrane potential enhances the binding affinity and reduce the leakage of cytochrome c to the cytoplasm^38^,^39^.

Glycolysis is tightly regulated at several stages and PFK is the most crucial control point in the glycolytic pathway. The enzymatic activity is tightly regulated by the ATP/AMP ratio, citrate, and fructose-2,6-bisphosphate. Intracellular calcium also plays an important role in the activity of PFK: high intracellular calcium leads to the binding of calmodulin to the high-affinity site of PFK monomer and stabilizes the PFK dimeric form with full activity^15^. MPTP-treated monkeys demonstrated a decrease of the enzyme activities related to the glycolytic pathway, especially the decrease in PFK^40^. It has been postulated that CMV infection increases glycolysis by activation of the calmodulin-dependent pathway^41^ and pUL37 × 1 is essential for this biogenergetic effect because it triggers the release of calcium storage from the ER^16^. In the present study, pUL37 × 1 over-expression did not alter the expression level of PFK and other glycolytic enzymes, but did increase PFK activity. Moreover, calcium caging by BAPTA-AM attenuated the increased glycolytic capacity of pUL37 × 1, which also supports the role of calcium-dependent increased glycolysis. It is known that alteration of intracellular Ca^2+^ is related to apoptosis. Also known is that Ca^2+^ content in ER determines the sensitivity to apoptotic stimuli^42^,^43^. Dysregulated Ca^2+^ signalling is considered as a contributing factor in the pathogenesis of PD^44^, in which the specific involvement of calpain has been investigated in toxin-induced PD models^45^,^46^,^47^. Any role of pUL37 × 1 in Ca^2+^ homeostasis remains uncertain.

The usage of two toxins, staurosporine and 6-OHDA in the present study, serves different purposes. Staurosporine is protein kinase c inhibitor and a strong apoptosis inducer as well. It triggers remarkable mitochondrial depolarization and Bax translocalization^48^. The protection of pUL37 × 1 against staurosporine-induced neuronal apoptotic death confirmed that pUL37 × 1 is able to be neuroprotective through anti-apoptosis. 6-OHDA is served to provide a PD *in vitro* model, which induced both apoptosis and necrosis though excessive oxidative stress^49^,^50^,^51^. pUL37 × 1 reduced not only caspase-3 activation but also total cell death, which reflect the summation of apoptosis and necrosis.

In conclusion, the mitochondrial mechanisms by which pUL37 × 1 inhibits apoptosis and prevents cell death may serve as a model for potential neuroprotective therapies that involve mitochondrial pathways.

## Material and Methods

### Cells and plasmids

SH-SY5Y cells were maintained as previous descriptions^52^ with reagents supplied from Life Technologies (Paisley, UK). The method for setting up E18 primary rat cortical culture was adapted from previous well-established protocols^53^, and they were used 7 days post-culture. p*ul37* × *1* open reading frame (coding sequence complement joining 50262..51197,51302..51344,52573..53060; Genbank accession NC_006273) added with a 3′ haemagluttinin (HA) epitope (total reading frame length 1494 nucleotides) was cloned into pcDNA3.1 plasmid. Stable ectopic expression was established in SH-SY5Y cells.

### Protein assay

Whole cell lysates for Western blot analysis were prepared in 10 mM Tris–HCl pH 7.5, 0.1% of SDS and in the presence of protease inhibitors (Fisher Scientific, Loughborough, UK), followed by DNAseI digestion (Promega, Southampton, UK). Protein samples were separated under reducing conditions by SDS-PAGE using the Novex system (Life Technologies), transferred to PVDF (Millipore, Watford, UK ) and were analyzed by a standard Western blot protocol using Amersham ECL Western Blotting Detection Reagent and Amersham Hyperfilm (GE Healthcare, Little Chalfont, UK). The following antibodies were used: anti-HA epitope antibody (clone 16B12, Life Technologies), anti-β-actin antibody (Abcam, Cambridge, UK), anti-Bax (NT) antibody (Millipore), anti-phosphofructokinase antibody, anti-pyruvate kinase antibody, and hexokinase 1 and 2 antibody (New England Biolabs, Hitchin, UK ). Protein concentration was decided by bicinchoninic acid assay (BCA) according to manufacturer’s instructions (Fisher Scientific).

### Immunocytochemistry and confocal microscopy

For immunocytochemistry, cells were fixed with 4% paraformaldehyde in phosphate buffered solution (PBS), permeabilized by adding cold methanol, and then processed in succession with a primary antibody, either anti-HA epitope antibody (Cambridge Bioscience, Cambridge,UK), anti-TOM20 antibody (Santa Cruz) or anti-Bax (NT) antibody (Millipore), Alexa Fluor secondary antibody (Life Technologies), and mounted with Citifluor (London, UK) in the presence of DAPI. Fluorescent images were obtained by epifluorescent microscope. In order to obtain image slices <1.5 μm, a Zeiss 510 laser scanning microscope was used.

### Apoptosis and cell death assay

Cytochrome c ELISA was performed according to manufacturer’s instructions (Life Technologies). Cells were lysed by a buffer containing 10 mM Tris, pH 7.4, 100 mM NaCl, 1 mM EDTA, 1 mM EGTA, and 10% glycerol incubated for 10 minutes. Cytosolic fraction was separated by a centrifugation at 12,000× g at 4 °C for 10 minutes. Caspase-3 activity was measured from cell lysates according to the manufacturer’s instruction of the EnzChek^®^ Caspase-3 Assay Kit #2 (Life Technologies) which has been described previously^54^. Apoptotic neurons in primary rat cortical culture were identified by anti-cleaved caspase-3 antibody (New England Biolabs). Apoptosis was induced by 20 μM 6-OHDA treatment for 6 hours and followed by the aforementioned immunocytochemistry protocols. LDH release assay (Roche, Burgess Hill, UK) was used to quantify the cell death and the protocols had been described^55^. PI staining was used to distinguish between live and dead cells. Both cell death measurements are based on similar principle: the ratio of LDH release/PI fluorescence between experiments to Triton X-100 treated control would be recognized as the percentage of cell death. For the detection of neuronal death in primary culture, cells were stained with 0.001% Fluoro-Jade C (Millipore) after fixed with 4% paraformaldehyde. Positive stained cells were counted under by epifluorescent microscope. The count of the death/apoptosis neurons is made under blinded condition. For each cover slips, 8–10 fields were selected. The number of RFP expressed cells with positive FJ-C or cleaved caspase-3 stain divided by the total number of RFP expressed cells was the percentage of death/apoptosis.

### siRNA silencing

10^5^ SH-SY5Y cells suspended in 500 μl growth medium were incubated with 6 nM siRNA (Dharmacon, GE Health) and 4.5 μl of HiPerFect transfection reagent (Qiagen, Manchester, UK) at 37 °C/5% CO_2_ for 24 hours. The efficiency of silencing was confirmed by Western blot analysis.

### Mitochondrial membrane potential measured by Tetramethylrhodamine, methyl ester(TMRM)

Steady stable mitochondrial TMRM (Life Technologies) fluorescence was measured from confocal images as previously described^56^,^57^. Images were analyzed by ImageJ software (National Institutes of Health, Maryland, U.S.) following protocols of analysis that have been previously described^58^.

### OCR and extracellular acidification rate (ECAR)

OCR and ECAR were measured by the XF extracellular flux analyzer following manufacturer’s instructions (Seahorse Bioscience, MA, US). For measuring OCR, 25 mM glucose, 1 μg/ml oligomycin and 2 μM rotenone were loading sequentially whereas for ECAR, 25 mM glucose, 1 μg/ml oligomycin and 100 μM 2DG were applied. 30 nM BAPTA-AM (Life Technologies) which was used to cage intracellular calcium was applied in ECAR measurement after glucose addition.

### PFK assay

PFK enzymatic activity was measured according to manufacturer’s protocols (Sigma–Aldrich, Dorset, UK).

### ADP phosphorylation ability and mitochondrial respiratory chain complex enzymatic activity measurement

The measurement of ADP phosphorylation ability and respiratory chain complex enzymatic activity had been described by Gegg *et al*.^59^. Enzymatic activity was measured by spectrophotometric methods and ADP phosphorylation was measured by ATP Bioluminesence Assay kit HSII.

### Intracellular and mitochondrial calcium measurement

Cells were incubated with 5 ng/ml of Fluo-4 FF, AM and Rhod-2, AM (Life Technologies) in standard HBSS in the presence of 0.0025% Pluronic for 30 minutes at room temperature, followed by another incubation in HBSS alone for another 30 minutes. Steady-state confocal images were obtained using a Zeiss 510 laser scanning microscope, to calculate the average net fluorescence after background subtraction. Fluo-4 and Rhod-2 were excited at 48 nm and 543 nm respectively.

### Statistics

All data were presented as mean ± standard error of mean (S.E.M.). Statistics was performed by either two-tailed Student’s t- test or one-way ANOVA with post-hoc analysis. *p* value less than 0.05 is recognized as significant.

## Additional Information

**How to cite this article**: Hong, C. T. *et al*. The Cytomegalovirus protein pUL37 × 1 targets mitochondria to mediate neuroprotection. *Sci. Rep.* **6**, 31373; doi: 10.1038/srep31373 (2016).

## Supplementary Material

Supplementary Information
