# Supplementary material for: The Cytomegalovirus protein pUL37×1 targets mitochondria to mediate neuroprotection
Source: Sci Rep. 2016 Aug 26;6:31373. doi: 10.1038/srep31373 (PMC4999870; doi:10.1038/srep31373)
Supplement: Supplementary Information [file srep31373-s1.doc]

**Title: The Cytomegalovirus Protein pUL37x1 targets mitochondria to mediate neuroprotection**

**Authors:**

Chien Tai Hong^1,2,3*^, Kai-Yin Chau^1,*^, Anthony HV Schapira^1, #^

**Affiliation:**

^1^Department of Clinical Neuroscience, Institute of Neurology, University College London, UK

^2^Department of Neurology, Shuang Ho Hospital, Taipei Medical University, New Taipei City, Taiwan

^3^Department of Neurology, School of Medicine, College of Medicine, Taipei Medical University, Taipei, Taiwan

^*^these authors contributed equally to this work

^#^Corresponding author

Proofs and reprint requests to: Anthony HV Schapira, 3B-94, Upper 3rd Floor

Institute of Neurology, Hampstead Campus. Rowland Hill St. London

NW3 2PF

e-mail: [a.schapira@ucl.ac.uk](mailto:a.schapira@ucl.ac.uk)

Tel: 020 7830 2012

Fax: 020 7472 6829

**Supplementary data**

S1. The protection of pUL37x1 over-expression by propidium iodide (PI) binding assay


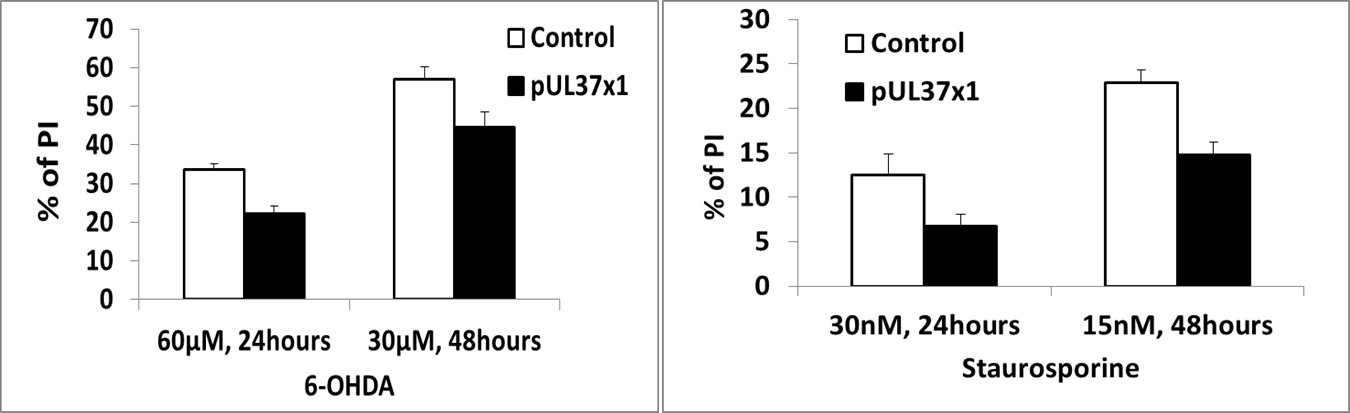


A

B

*

***

*

***

Figure S1(A). pUL37x1 over-expression significantly decreased the percentage of PI fluorescence induced by 60 μM 6-OHDA treatment for 24hours and 30μM 6-OHDA treatment for 48hours (60 μM 6-OHDA, 24hours: 33.6±1.5% in control, 22.2±2.0% in pUL37x1 over-expression, p<0.001, n=6; 30μM 6-OHDA, 48hours: 56.9±3.4% in control, 44.7±4.0% in pUL37x1 over-expression, p<0.05. n=6). (B) pUL37x1 over-expression reduced the PI fluorescence induced by staurosporine. The protection was noted in either 15nM treatment for 48 hours or 30nM treatment for 24 hours (15nM treatment for 48 hours: 22.9±1.4% in control, 14.7±1.5% in pUL37x1 over-expression, p<0.001, n=8; 30nM treatment for 24 hours: 12.5±2.4% in control, 6.8±1.3% in pUL37x1 over-expression, p<0.05, n=6). Data were presented as mean±S.E.M. Statistics was performed by two-tailed Student’s t- test. The n number represented the experimental repeat from each pUL37x1 over-expression lines and control (normal SH-SY5Y cells, and over-expressing pcDNA Zero SH-SY5Y cells) (*,p<0.05, ***, p<0.001).

S2. The protection of pUL37x1 over-expression from individual line

A


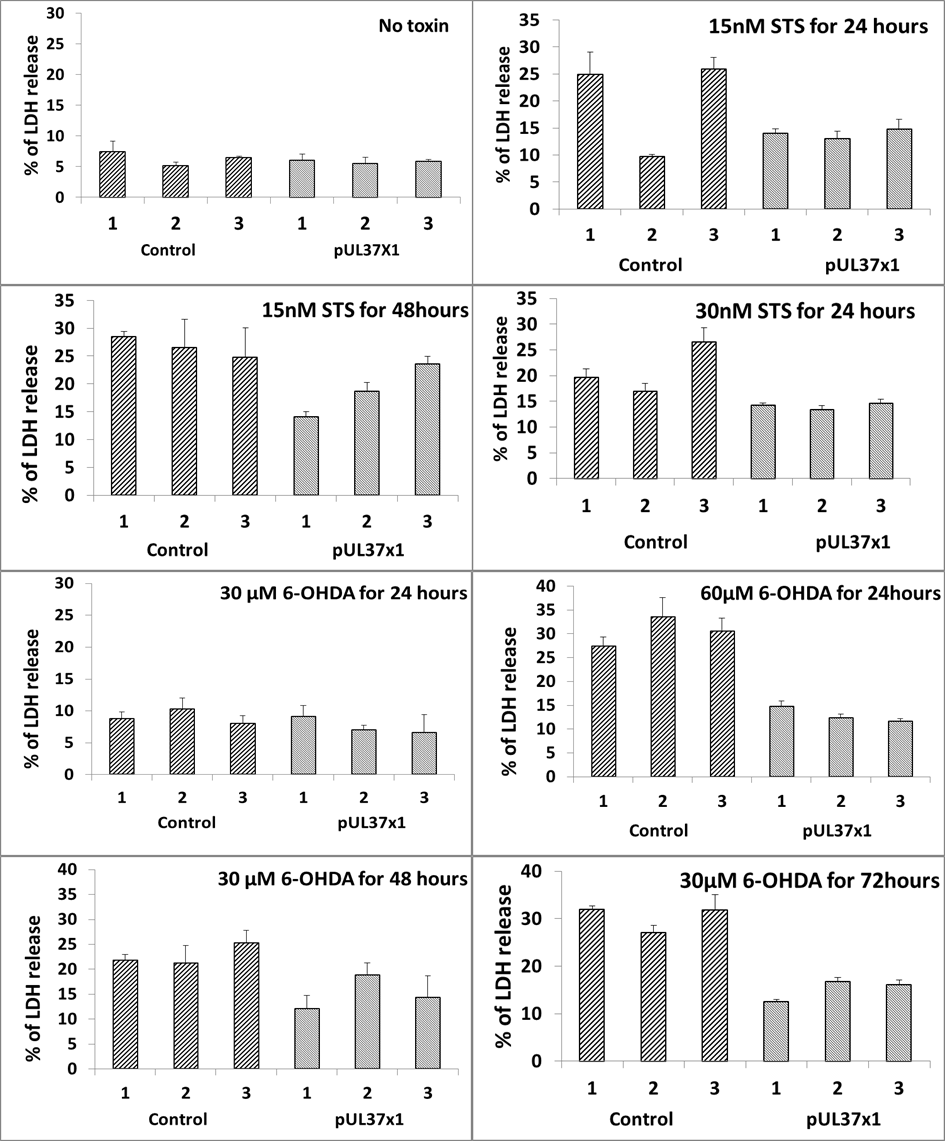


B

C

D

E

F

G

H

**

**

*

**

*

***

***

***

***

***

***

***

***

***

Figure S2. (A) pUL37x1 over-expressing SH-SY5Y lines did not increase spontaneous cell death in no-toxin status compared with control. (B) pUL37x1 over-expression reduced the percentage of LDH release against 15nM staurosporine treatment for 24 hours compared with control SH-SY5Y cells (control-1). All three pUL37x1 over-expressing line exhibited significant protection against staurosporine. (C) pUL37x1 over-expression reduced the percentage of LDH release against 15nM staurosporine treatment for 48 hours compared with control SH-SY5Y cells (control-1). pUL37x1-1 over-expressing line exhibited significant protection against staurosporine. (D) pUL37x1 over-expression reduced the percentage of LDH release against 30nM staurosporine treatment for 24 hours compared with control SH-SY5Y cells (control-1). All pUL37x1 over-expressing lines exhibited significant protection against staurosporine. (E) 30µM 6-OHDA treatment for 24 hours caused less than 10% of LDH release in SH-SY5Y cells (control-1). There was no significant difference between control and pUL37x1 over-expressing cells in the percentage of LDH release. (F) pUL37x1 over-expression reduced the percentage of LDH release compared with control SH-SY5Y cells (control-1). All three lines exhibited significant protection against 6-OHDA. (G) pUL37x1 over-expression reduced the percentage of LDH release against 30µM 6-OHDA treatment for 48 hours compared with SH-SY5Y cells (control-1). (H) pUL37x1 over-expression reduced the percentage of LDH release against 30µM 6-OHDA treatment for 72 hours compared with control SH-SY5Y cells (control-1). All three lines exhibited significant protection against 6-OHDA. Data were presented as mean±S.E.M. Statistics was performed by one-way ANOVA with Dunnett’s post-hoc analysis. ( *, p<0.05, **, p<0.01,***, p<0.001)

S3. pUL37x1 over-expression induced Bax tranlocation to mitochondria and sliencing of *bax* in control and pUL37x1 over-expressing SH-SY5Y cells

A


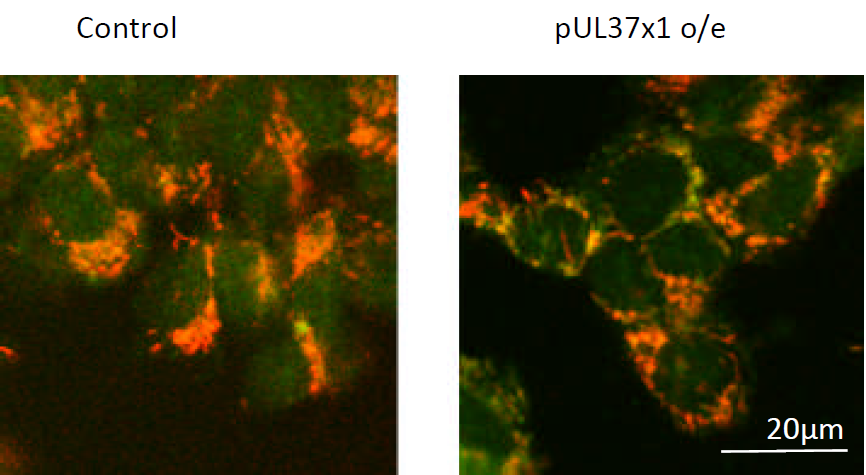


B

(A) pUL37x1 over-expression induced the mitochondrial translocation of Bax. In control, Bax (green) demonstrated a predominant intracellular distribution with scant co-localization with MitoTracker (red). Nevertheless, in pUL37x1 over-expressing ( pUL37x1 o/e) SH-SY5Y cells, a remarkable co-localization with Bax and MitoTracker was shown (yellow), which indicated a translocation of Bax from cytoplasm to mitochondria. Images were obtained from confocal microscope at <1.5μm.

(B) Representative Western blot image demonstrated that 5nM *bax* siRNA silencing for 3 days remarkably lowered the Bax protein level in both normal and pUL37x1 over-expressing SH-SY5Y cells compared with scramble siRNA (Scr) silencing.

S4. pUL37x1 over-expression did not affect the ATP synthesis and mitochondrial respiratory chain activity


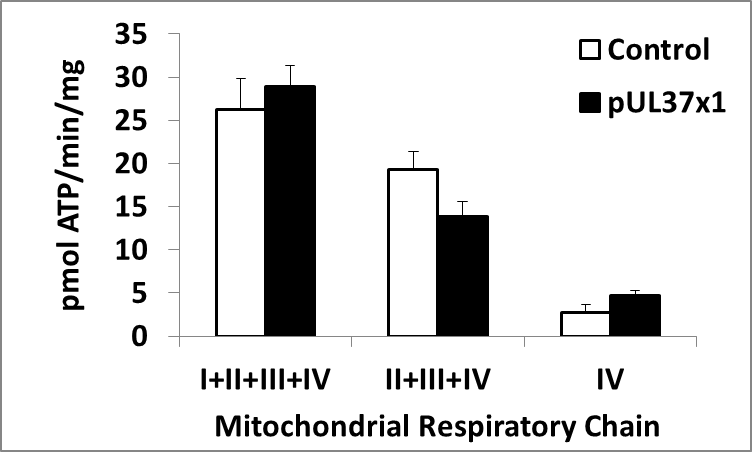


N.S.

N.S.

N.S.

| **Mitochondrial Respiratory Chain activity**  **(mmole/min/mg mitochondria)** | | | |
| --- | --- | --- | --- |
|  | **Complex I** | **Complex II+III** | **Complex IV** |
| **Control** | **0.68±0.06** | **0.20±0.02** | **0.009±0.0007** |
| **pUL37x1** | **0.81±0.06** | **0.24±0.02** | **0.012±0.0007** |

pUL37x1 over-expression did not affect mitochondrial ADP phosphorylation ability and mitochondrial respiratory chain activity from each respiratory chain complex. In the figure, the ADP phosphorylation ability was no significant different between control and pUL37x1 over-expressing lines (control: complex I+II+III+IV: 26.3±3.6, complex II+III+IV: 19.3±2.0, complex IV: 2.7±0.9; pUL37x1: complex I+II+III+IV: 28.9±2.5, complex II+III+IV: 13.9±1.7, complex IV: 4.6±0.6 pmole ATP/min/mg, p>0.05 in each condition, n=3). The table demonstrated that the enzymatic activity of each mitochondrial respiratory chain complex is not different between control and pUL37x1 over-expressing lines (control: complex I: 0.68±0.06, complex II+III: 0.20±0.02, complex IV: 0.009±0.0007; pUL37x1 over-expressing cells: complex I: 0.81±0.06, complex II+III: 0.24±0.02, complex IV: 0.012±0.0007 mmole/min/mg mitochondria, n=3) . The n number represented the experimental repeat from each pUL37x1 over-expressing lines and control SH-SY5Y cells. (NS, non-significant).
